# The Expression of and Preoperative Correlation between Heat-Shock Protein 70, EuroSCORE, and Lactate in Patients undergoing CABG with Cardiopulmonary Bypass

**DOI:** 10.21470/1678-9741-2018-0231

**Published:** 2019

**Authors:** Marcos Antonio Cantero, Rui Manuel Siqueira Almeida, Priscila Neder Morato, Valfredo de Almeida Santos-Junior, Carolina Soares Moura, Jaime Amaya-Farfan, João Luis Fonseca, Pablo Christiano Barboza Lollo

**Affiliations:** 1 Faculdade de Ciências da Saúde, Universidade Federal da Grande Dourados, Dourados, MS, Brazil.; 2 Department of Cardiology and Cardiovascular Surgery, Universidade Estadual do Oeste do Paraná, Cascavel, PR, Brazil.; 3 Faculdade de Engenharia de Alimentos, Universidade Estadual de Campinas, Campinas, SP, Brazil.

**Keywords:** HSP 70 Heat-Shock Proteins/Blood, Heat-Shock Proteins, Coronary Artery Bypass, Cardiopulmonary Bypass

## Abstract

**Objetive:**

Coronary artery bypass grafting (CABG) with cardiopulmonary bypass (CPB) improved symptoms and increased survival and quality of life in patients with coronary artery disease. However, it should be the main cause of a complex organic systemic inflammatory response that greatly contributes to several postoperative adverse effects.

**Methods:**

We aimed to evaluate heat-shock protein 70 (HSP 70) expression as a morbimortality predictor in patients with preserved ventricular function undergoing coronary artery bypass grafting (CABG) with cardiopulmonary bypass (CPB) and to determine their association with the lactate as a marker of tissue hypoperfusion and the EuroSCORE risk score. This is a prospective, observational study including 46 patients and occurring between May and July 2016. Patients without ventricular dysfunction undergoing myocardial revascularization with extracorporeal circulation were included. They were divided into (1) complicated and (2) uncomplicated postoperative evolution groups. EuroSCORE, lactate levels, and HSP 70 expression and their correlations were determined.

**Results:**

Statistical analysis showed that the group with complicated evolution had higher EuroSCORE values than the other group. HSP 70 protein levels were significantly increased in the group with uncomplicated evolution and showed similar results. According to our results, HSP family proteins may be independent predictors of uncomplicated evolution in patients without ventricular dysfunction undergoing CABG with CPB.

**Conclusion:**

HSP 70 should be a good discriminator and protection marker for complications in cardiac surgery.

**Table:** 

Abbreviations, acronyms & symbols				
ACT	= Activated coagulation time		ICU	= Intensive care unit
ATP	= Adenosine triphosphate		IR	= Interquartile range
BSA	= Body surface area		LL	= Lower limit
C	= Complicated		mRNA	= Messenger ribonucleic acid
CABG	= Coronary artery bypass grafting		NC	= Non-complicated
CI	= Confidence interval		OR	= Odds ratio
CO_2_	= Carbon dioxide		PMSF	= Phenylmethanesulfonyl fluoride
CPB	= Cardiopulmonary bypass		ROC	= Receiver operating characteristic
EDTA	= Ethylenediaminetetraacetic acid		UFGD	= Federal University of Grande Dourados
Hb	= Hemoglobin		UL	= Upper limit
HSP	= Heat-shock proteins		UNICAMP	= University of Campinas
HSP 70	= Heat-shock protein		VF	= Ventricular fibrillation
IAB	70 = Intra-aortic balloon			

## INTRODUCTION

Coronary heart disease is the main cause of mortality in the global population^[1]^. In recent decades, coronary artery bypass grafting (CABG) with cardiopulmonary bypass (CPB) have improved symptoms and increased survival rate and quality of life^[2]^. However, this surgical method is considered a cause of a complex organ system inflammatory response that contributes to several postoperative adverse effects, which include renal, pulmonary, neurological, and hemorrhagic complications^[3]^. In spite of significant changes and improvements in CPB systems, complications involving tissue damage persist, affecting postoperative morbidity and mortality^[4]^. Complications caused by CPB, such as bleeding, hemodilution, and low cardiac output, are related to tissue hypoxia and organ dysfunctions^[5,6]^.

To assess morbidity and mortality during cardiac surgery, several models of risk stratification have been developed^[3,7,8]^. EuroSCORE is a simple, objective, effective, and safe instrument even when applied to non-European populations^[9]^. In Brazil, it is used as postoperative morbidity and mortality predictor^[9,10]^. However, because there are multiple and complex intraoperative and postoperative factors involved and the prognosis needs to be safely assessed, it is necessary to evaluate tissue perfusion by ideally simple and accessible methods that can provide information to complement the EuroSCORE.

Lactate is widely used as a systemic indicator to evaluate the metabolism. Nonetheless, its clinical interpretation should be cautious because although it assesses the severity of the clinical situation, it is nonspecific, and the isolated measurement does not benefit the prognostic evaluation^[11]^. Serial measurements and comparison of their values provide a significant understanding of the case's severity^[12]^.

Heat-shock proteins (HSP) belong to the chaperone family of proteins present in the body cells. They correspond to approximately 1%-2% of the constituent proteins in non-stressed cells and 4%-6% of the proteins in stressed cells. High molecular weight HSP (HSP 100, 90, 70, and 60) are adenosine triphosphate (ATP)-dependent, whereas low molecular weight HSP (HSP 20, 25, and 27) are ATP-independent^[13]^. They provide the cell with great tolerance and resistance against a variety of aggressor agents, maintaining the cell's integrity and structure, besides promoting cell survival during stress periods^[14]^.

Because HSP can respond to different types of stress, which include heart disease, its potential as a diagnostic and prognostic marker in heart disease has been proposed^[15,16]^.

There is a great interest in understanding the role of HSP in the inflammatory response that occurs during cardiac surgery with CPB. Several studies have demonstrated an increase in the production of pro-inflammatory cytokines after CPB^[17]^.

Among the HSP types, HSP 70 is the most studied intracellular chaperone molecule. Many efforts have been directed at assessing its capacity for protecting the cardiac tissue undergoing ischemic preconditioning^[18]^. Experimental results suggest that the effect of moderate hypothermia during CPB involves upregulation of HSP 70 with inhibition of necrosis, but not of apoptosis^[19]^. HSP 70 levels are associated with tissue damage and ischemia following cardiac surgery and their measurement may present a diagnostic and prognostic advantage in these cases^[20]^.

Therefore, we aimed to evaluate HSP 70 expression as a morbimortality predictor in patients with preserved ventricular function undergoing CABG with CPB and to determine their association with the lactate as a marker of tissue hypoperfusion and EuroSCORE risk score.

## METHODS

### Study Design and Protocol

The study protocol was evaluated and approved by the Graduate Teaching and Research Committee of the Federal University of Grande Dourados (UFGD) and the Ethics Committee for Analysis of Research Projects on Human Subjects of the University of Campinas (UNICAMP). The protocol was registered on the 'Plataforma Brasil' research website (CAAE Registry No. 50344015.8.3001.5404). A prospective, observational study was conducted between May and July 2016 at the Hospital Evangélico Dr. e Sra. Goldsbyking (Dourados, MS, Brazil).

The patients were then divided into two groups, according to their postoperative evolution: (1) patients with complicated evolution and (2) those with uncomplicated evolution. Complicated evolution was defined as death during hospitalization or in the first 30 days after surgery and/or the presence of one or more postoperative complications that resulted in prolonged intensive care unit (ICU) hospitalization (length of stay > 4 days). All other cases were considered uncomplicated. The post-surgery outcomes were compared between the groups.

### Patients' Recruitment

During the study period, 93 patients scheduled to undergo cardiac surgery with CPB in the institution were selected. After applying the exclusion criteria, 52 patients were considered eligible for the study. Among them, two patients did not sign the informed consent form and four patients were excluded due to data loss. Thus, 46 patients were included. The study inclusion criteria included adult patients without left ventricular dysfunction who were scheduled to undergo CABG with CPB. All subjects gave written informed consent to participate in the study and were informed (orally and in detail) by the researchers about the nature, investigative character, objectives, results, and risks of the study.

The exclusion criteria comprehended patients with chronic or acute renal failure (serum creatinine levels ≥ 1.4 g/dL), hepatic failure, uncontrolled diabetes mellitus, pulmonary diseases (chronic obstructive pulmonary disease or previous pulmonary surgery), neurological diseases (stroke, dementia, or psychosis), history of fever or recent infection (up to 1 week prior to surgery), presence of cardiogenic shock or mechanical complications of infarction, abnormal ejection fraction (< 55%), and non-compliance with the method on the part of the patient.

### Surgery Protocol

The standard pre-anesthetic medication was 1 mg alprazolam, administered orally 8 hours before surgery; no medication was administered to patients with unstable hemodynamic conditions. The anesthetic technique chosen was balanced general anesthesia. After denitrogenation with 100% oxygen, anesthetic induction was performed with etomidate (2 mg/kg), midazolam (0.05 mg/g), sufentanil (0.5 µg/kg), or fentanyl (5 µg/kg), and pancuronium (0.1 mg/kg) was used as a neuromuscular blocker. The patients were then intubated and maintained on mechanical ventilation with a fraction of inspired oxygen of 60% in the valvular circulatory system with carbon dioxide (CO_2_) absorber, volume-controlled and pressure-limited ventilation (25 mmHg), tidal volume ranging from 6 to 8 mL/kg, respiratory rate of 12 cycles per minute, positive end-expiratory pressure of 5 mmHg, and gas flow of 1.0 L/min. Anesthesia was maintained using varying isoflurane concentrations (0.5%-1%) and intermittent doses of sufentanil or fentanyl and pancuronium. Before CPB, a new aliquot of midazolam and a neuromuscular blocker were administered.

Intraoperative hydration was performed with Ringer's lactate based on filling pressures, diuresis, and cardiac output. The red blood cell transfusion threshold was established as hemoglobin (Hb) < 7 mg/dL, in patients without hemodynamic instability, and Hb < 9 mg/dL, in those with hemodynamic instability.

Before CPB, patients underwent anticoagulation therapy with heparin (Liquemine®) at a dose of 300 IU/kg to maintain activated coagulation time (ACT) > 480 s, supplemented with doses of 5,000-10,000 IU when necessary (ACT < 480 s). At the end of CPB, anticoagulation was reversed with protamine hydrochloride at a 1:1 ratio of the initial dose. Patients underwent median sternotomy; CPB was initiated with cannulation of the ascending aorta and right atrium, and cannulation of the right upper pulmonary vein was initiated with the introduction of a catheter for aspiration and decompression of the left ventricle. Membrane oxygenators were used in all patients during CPB. The temperature during CPB was maintained between 32°C and 34°C, and the myocardial protection technique used was intermittent hypothermic antegrade cardioplegia. The management of the acid-base balance during CPB was performed using the alpha-stat method. Infusion was maintained with non-pulsatile flow of approximately 2.2-2.4 L/min/m to maintain a mean blood pressure of approximately 60 mmHg.

To remove the patient from CPB, vasoactive and inotropic drugs were used according to the patient's needs; this decision was left to the discretion of the anesthesiologist.

### Outcomes

The following preoperative clinical data were recorded: EuroSCORE; age; gender; weight; body surface area (BSA); left ventricular ejection fraction; history of previous cardiac surgeries; and use of intra-aortic balloon (IAB), inotropic and/or vasopressor drugs, and nitroglycerin.

During surgery, the patient's temperature, heart rate, cardiac rhythm, urinary output, arterial blood pressure (through the radial artery), and central venous pressure (through the subclavian vein) were measured. Intraoperative data were collected in the operating room, and postoperative data were collected in the surgical ICU of the same institution. After surgery, patients were monitored until discharge, and postoperative complications during the hospitalization period were recorded.

The following postoperative complications were observed: prolonged hospital stay (defined as hospital discharge after the 10th postoperative day), prolonged ICU stay (defined as ICU discharge after the 4th postoperative day), prolonged tracheal intubation time (defined as mechanical ventilation for more than 6 hours after admission to ICU or need for tracheal reintubation), neurological complications (focal or global neurological deficit documented within 48 hours after surgery or delirium), infectious complications (surgical wound infection, mediastinitis, pneumonia, urinary tract infection, and/or sepsis with no identified focus, according to the standards established by the Hospital Infection Control Committee), low cardiac output (defined as a cardiac index < 2.2 L/min/m^2^, difficulty in weaning from inotropic medications 24 hours after surgery, or the need for IAB use), arrhythmias (presence of arrhythmia requiring the use of antiarrhythmic agents and/or the need for defibrillation or electrical cardioversion), and acute kidney failure (defined as an increase in serum creatinine levels > 50% of the preoperative value or the need for hemodialysis).

Serum lactate levels were analyzed by arterial gasometry using the potentiometric method, with a reference value of 5.7-2.0 mg/dL or 0.63-2.44 mmol/L. The equipment used for the measurements was the Radiometer® ABL 700 gasometer.

### Western Blot Analysis

Myocardial biopsy for HSP assessment was performed on the free wall of the right atrium. The sample was obtained immediately after pericardial opening, prior to the administration of heparin and/or any surgical manipulation of the heart. Samples were preserved in liquid nitrogen and stored at -70°C until analysis. The frozen samples were homogenized in 20 volumes of 600 mmol/L sodium chloride and 15 mmol/L Tris (hydroxymethyl aminomethane); pH was 7.5. Protein levels were determined using the technique described by Lowry et al.^[21]^ and bovine serum albumin was used as a standard.

From each patient selected in this study, a 1 mL serum sample was collected during atrial biopsy for assessing circulating HSP 70 levels. Next, 1 µL of each protease inhibitor (phenylmethanesulfonyl fluoride [PMSF], aprotinin, leupeptin) was added to each plasma aliquot prior to freezing in a -80°C freezer. HSP 70 levels in the culture medium were determined using an ultra-sensitive specific kit (HSP70 EKS-715), and the result was normalized by the number of cells in each one and the incubation period, expressed as ng/h/108 cells.

The Western blot test was conducted as follows: the atrial muscle (200 mg) was homogenized in 1 mL of buffer (200 mM ethylenediaminetetraacetic acid [EDTA] [Sigma 03685], pH 7.0), 1 M Tris Base (Bio-Rad # 161-0719, pH 7.5), 10 mM orthovanadate (Sigma S6508), 2 mM PMSF (Sigma P7626), 10 mM sodium pyrophosphate (Sigma 221368), 0.1 mg/mL aprotinin (Sigma 10820), 100 mM sodium fluoride (Sigma 71519), 10% Triton (Sigma # 019K0151), and ultrapure water. The test used a polytron (Pro Scientific model 200) and centrifuged the solution (Sigma, 2K15 model, serial number 57707, Germany) at 14,000 g for 40 min at 4°C; at this point, the supernatant was collected. The total protein content in the supernatant was determined using the Lowry method^[21]^.

### Statistical Analysis

In the statistical analysis, continuous variables were presented as means ± standard deviations or as medians and interquartile ranges when data did not follow a Gaussian distribution. Categorical variables were presented as proportions.

Continuous variables were analyzed using Student's t-test or non-parametric Mann-Whitney test; categorical variables were analyzed using Fisher's exact test or the chi-squared test, when appropriate. Parameters that presented a difference between the groups with *P*<0.05 in the univariate analysis were included in the multivariate analysis, which was performed using a logistic regression model (backward Wald) to identify independent markers for complicated evolution. The logistic regression results were described as *odds ratio* (OR) and at a 95% confidence interval (95% CI).

Receiver operating characteristic (ROC) curves of parameters identified as complicated evolution predictors were constructed to find the best cut-off points associated with complicated evolution. The cut-off point was determined as the value associated with the highest sum of sensitivity and specificity. Areas under the ROC curve were determined and compared. The analysis was performed using the Statistical Package for Social Sciences software, version 17.0. A *P*-value < 0.05 was considered statistically significant.

## RESULTS

Of the 46 patients studied, 32 were men (69.5%) and 14 were women (30.4%). The ages of the patients ranged from 35-83 years old. The general characteristics were presented in Table 1. The mean age in the group of patients with complications was 66.3 ± 10 years while the mean age in the group of those without complications was 59.7 ± 9.4 years (*P*=0.002). Although a different value was observed, there was no statistically significant difference in BSA or in the proportion of female patients between the groups.

**Table 1:** General characteristics of the patients' groups.

Variable	Uncomplicated evolution (n = 31)	Complicated evolution (n = 15)	*P*	Test
Age (years)^a^	59.7±9.4	66.3±10	0.002	Student's t-test
BSA (m^2^)^a^	1.81±0.17	1.71±0.16	0.06	Student's t-test
Weight (kg)^b^	74 (65.5-80)	65 (60-73.5)	0.005	Mann-Whitney
Female gender^c^	8 (25.8%)	6 (40%)	0.13	Chi-squared
Ejection fraction (VF)	58±1.3	56±1.2	0.25	Mann-Whitney

a Values A1:E18 mean ± standard deviation;

b median values and interquartile range;

c absolute value (percentages)

BSA=body surface area; VF=ventricular fibrillation

The group with complicated evolution presented higher EuroSCORE values (median, 5; interquartile range [IR], 3-5) than the group with uncomplicated evolution (median, 3; IR, 3-5). No differences were observed between the groups with respect to surgery, anesthesia, aortic clamping, and CPB duration (Table 2).

**Table 2:** Preoperative variables of patients.

Variable	Uncomplicated evolution (n=31)	Complicated evolution (n=15)	*P*	Test
EuroSCORE^b^	3 (3-5)	6 (5-8)	<0.001	Mann-Whitney
IAB^c^	3 (6.1%)	4 (10.5%)	0.71	Fisher's test
Inotropic and/or vasopressor drugs^c^	1 (3.0%)	3 (20%)	0.44	Fisher's test
Nitroglycerin^c^	4 (13%)	2 (12.5%)	0.92	Fisher's test
Intraoperative				
Surgery duration	280 (240-340)	312 (245-374)	0.24	Mann-Whitney
Anesthesia duration	390 (320-430)	400 (334-480)	0.38	Mann-Whitney
CPB time (min)^b^	95 (75-118)	90 (69-140)	0.77	Mann-Whitney

b Median values and interquartile range;

c absolute value (percentages)

CPB=cardiopulmonary bypass; IAB=intra-aortic balloon

During the postoperative follow-up, 31 patients were classified as having uncomplicated evolution (67.3%) and 15 patients had complicated evolution (32.6%), three of whom died within 30 days of surgery, one of neurological complication and two of infectious complications (Table 3). The other postoperative parameters that configure the differences between the groups were presented in Table 3.

**Table 3:** Comparison between postoperative data of patients who underwent CABG with CPB with uncomplicated and complicated evolutions.

Variable	Uncomplicated evolution (n=31)	Complicated evolution (n=15)	*P*
Duration of ICU stay (days)^a^	2 (2-4)	8 (7-21)	<0.001
In-hospital length of stay (days)^a^	7 (6-13)	21 (14-28)	<0.001
Prolonged intubation^b^	18 (41%)	11 (75%)	<0.001
Renal complications^b^	1 (3%)	8 (51%)	<0.001
Neurological complications^b^	0 (0%)	4 (26%)	<0.001
Infectious complications^b^	9 (29%)	11 (73%)	<0.001
Low cardiac output^b^	3 (9.6%)	13 (86.6%)	<0.001
Arrhythmias^b^	2 (6.4%)	9 (60%)	<0.001

a Values in median and interquartile range;

b Absolute value (percentage)

CABG=coronary artery bypass grafting; CPB=cardiopulmonary bypass; ICU=intensive care unit

**Fig. 1:**
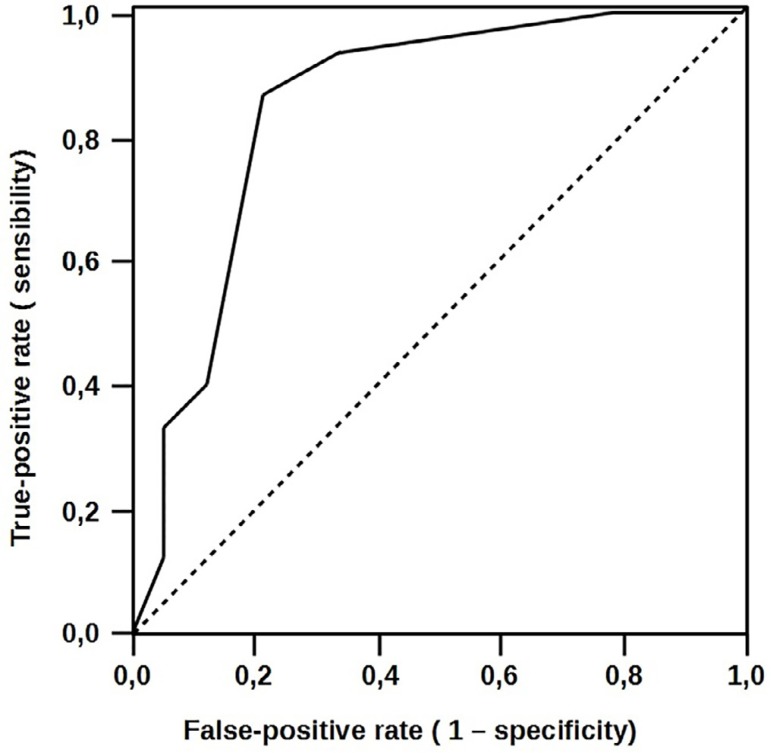
EuroSCORE receiver operating characteristic (ROC) curves to predict complications.

The area under the curve was 0.841 (95% CI: 0.725-0.956). This indicates that the EuroSCORE has a high power to predict complications. The chance that a patient will present complications increases by 2.09 times with each additional EuroSCORE point. This is a strong independent risk and prognosis predictor, as shown in Figure 1.

**Fig. 2:**
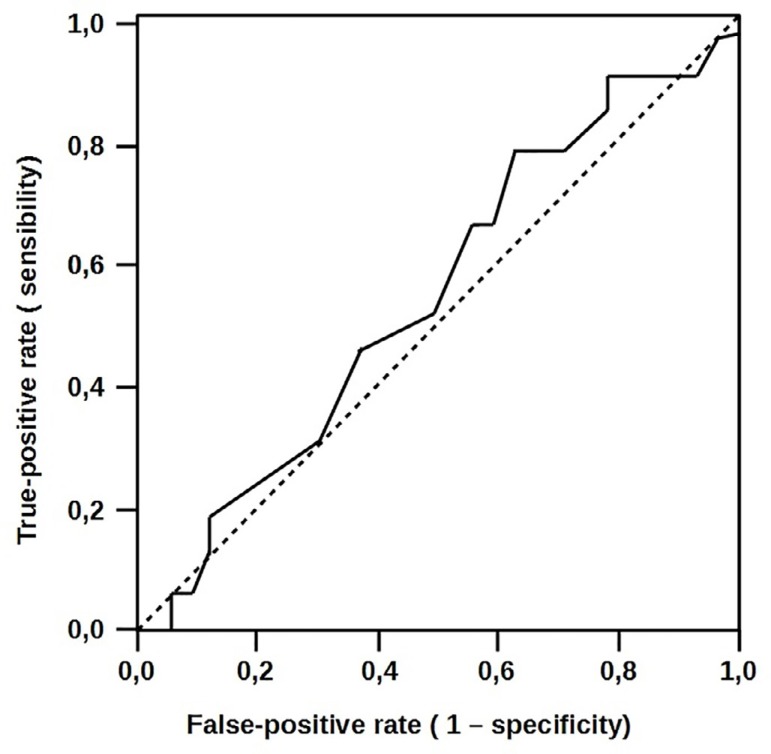
Lactate receiver operating characteristic (ROC) curves to predict complications.

Arterial lactate levels were significantly higher in the group with complicated evolution than in the other group. Baseline levels of lactate were 452 ± 88 in the uncomplicated group and 510 ± 105 µM in the complicated group (*P*=0.40). Despite being increased in the complicated group, the delta lactate showed no significant difference in lactate levels between the groups. The results indicate that there was a significant increase in arterial lactate levels during surgery in both groups, but this was not an independent predictor of complications. As shown in Figure 2, the area under the curve was 0.558 (95% CI: 0.386-0.73), and its accuracy was not significant in this sample.

**Fig. 3:**
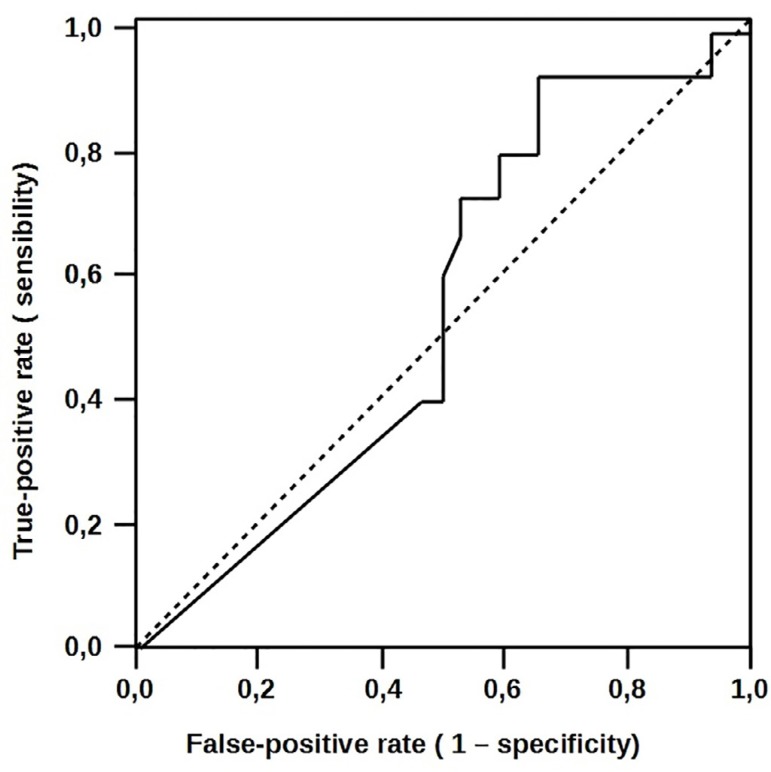
Atrial heat-shock protein 70 (HSP 70) expressions receiver operating characteristic (ROC) curves to predict complications.

**Fig. 4:**
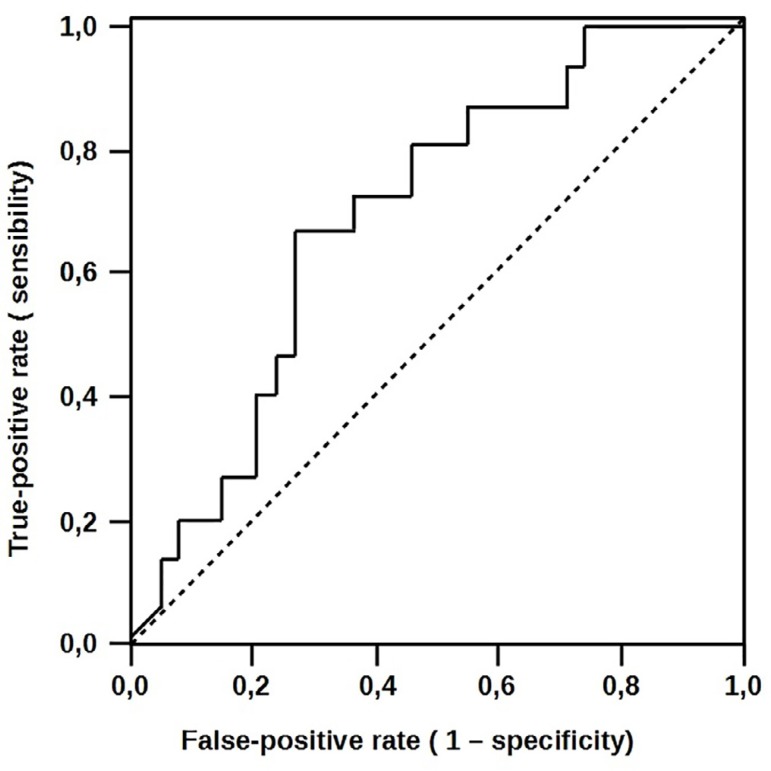
Serum heat-shock protein 70 (HSP 70) receiver operating characteristic (ROC) curves to predict complications.

The significant increase in HSP 70 expression was observed in the group with uncomplicated evolution. HSP 70 content was significantly higher in patients who did not develop complication (34±12 *vs*. 18±14; *P*=0.006). HSP 70 area under the curve was 0.683 (95% CI: 528-0.839), with a sensitivity of 93% of protection. These results held true even in peripheral plasma (37±12 *vs*. 22±15, *P*=0.01). HSP 70 presented the same expression in the peripheral plasma with an area under the curve of 0.687 (95% CI: 0.377-0.715) and a significant sensitivity of 93%, as observed in Figures 3 and 4, respectively.

**Fig. 5:**
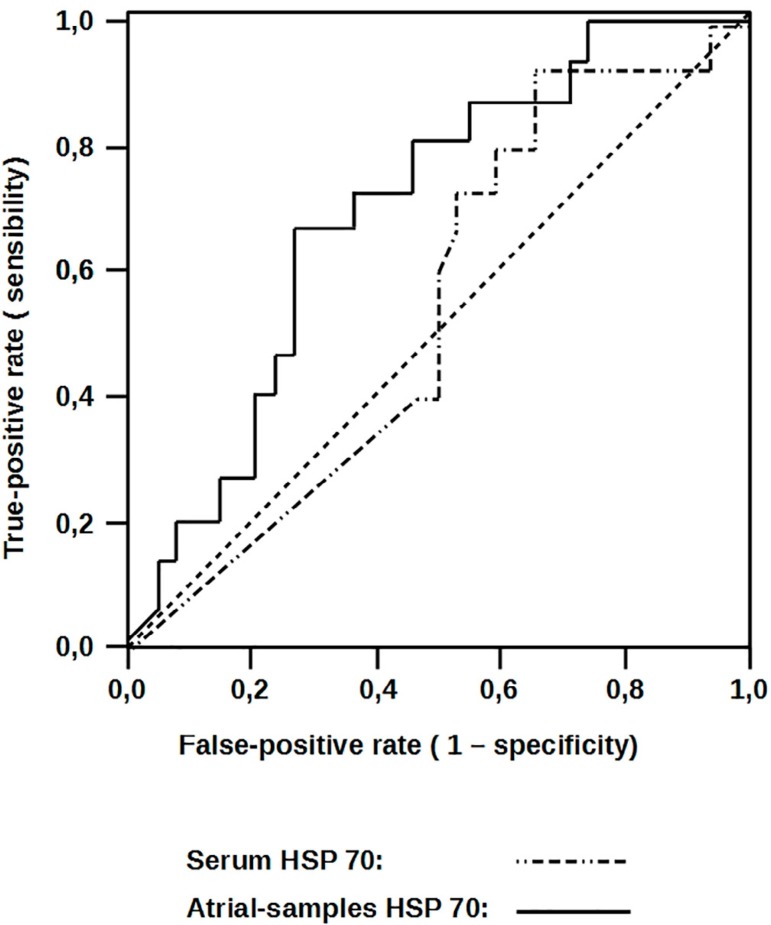
Serum and atrial heat-shock protein 70 (HSP 70) receiver operating characteristic (ROC) curves to predict complications.

In our study, atrial biopsy was obtained immediately after opening the pericardium, before administration of heparin-initiation of CPB. Inducible HSP 70 level in such relatively unstressed atrial myocytes reflects initial expression of HSP 70 and possibly represents a more efficient stress handling mechanism at the disposal of these cells. Soluble HSP 70 concentrations may be a marker of cell injury induced by severe stress and are not reflective of intracellular levels; this is the difference in ROC curve for atrial *vs*. serum HSP 70 in Figure 5.

From the univariate CI, it was observed that variable lactate levels were not risk factors for patients with complications. Areas under the ROC curve demonstrate that EuroSCORE and HSP 70 variables were protective factors for the occurrence of complications. These findings indicate that EuroSCORE and HSP 70 variables presented excellent discrimination power, as observed in Table 4.

**Table 4:** Statistical analysis of the markers studied.

Variable	Univariate regression (95% CI)				Multivariate regression (95% CI)			
	OR	LL	UL	*P*-value	OR	LL	UL	*P*-value
EuroSCORE	2.097	1.326	3.316	0.002	1.99	1.259	3.147	0.003
Lactate	1.573	0.978	2.529	0.062	1.449	0.744	2.82	0.276
Atrial HSP 70	0.787	0.666	0.929	0.005	1.146	0.71	1.85	0.577
Serum HSP 70	0.71	0.501	1.004	0.053	1.25	0.704	2.219	0.446

CI=confidence interval; HSP 70=heat-shock protein 70; LL=lower limit; OR=odds ratio; UL=upper limit

**Fig. 6:**
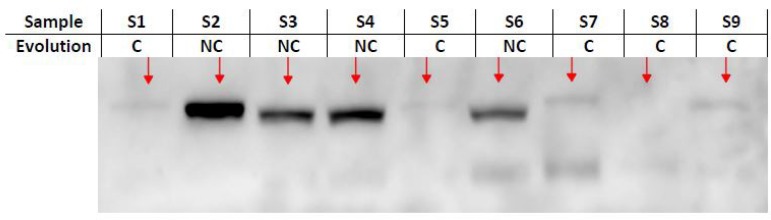
Myocardial expression of heat-shock protein 70 (HSP 70) and evolution outcome. C=complicated; NC=non-complicated

**Fig. 7:**
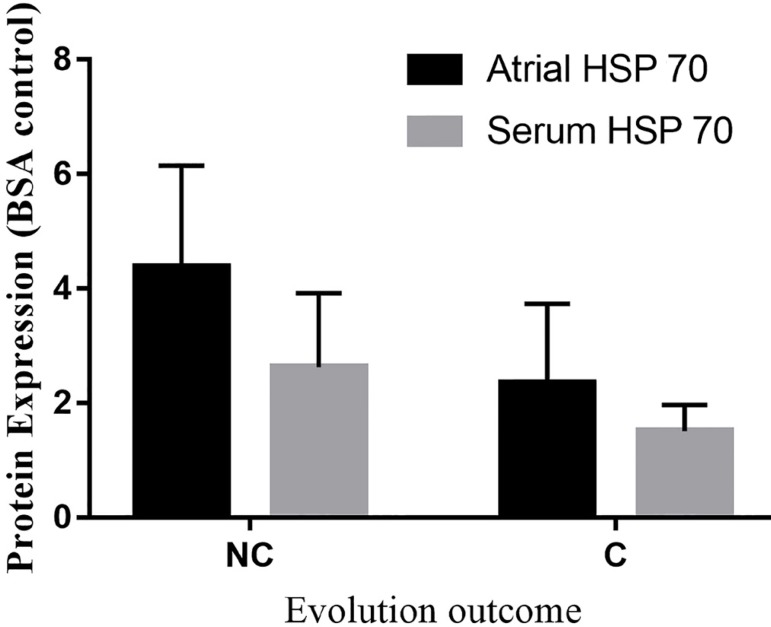
Atrial and serum heat-shock protein 70 (HSP 70) expression in the complicated (C) and non-complicated (NC) group. BSA=body surface area

The qualitative expression of HSP 70 showed that the level of protein was correlated to the outcome of post-surgery evolution, as shown in the Figure 6. The non-complicated group showed higher bands expressions of serum and atrial HSP 70 levels (Figure 7) than the complicated group.

**Fig. 8:**
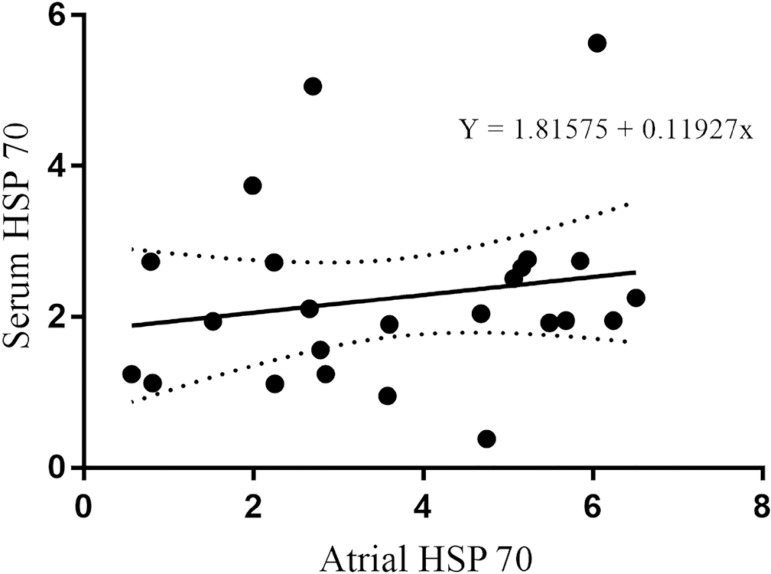
Linear regression between serum and atrial heat-shock protein 70 (HSP) 70 expression.

The dispersion plot of correlation between serum and atrial HSP 70 expression showed a moderate and non-significant coefficient of relationship (*P*>0.05) (Figure 8). Linear regression equation is presented in the Figure 8.

## DISCUSSION

CABG with CPB is a highly effective treatment for coronary insufficiency; however, it has a non-negligible potential for complications. The identification of markers of these complications can minimize or even prevent them and the HSP 70 expression seems to be a good way to measure the complication risk. This study evaluated the expression of HSP 70 in the myocardium and peripheral blood and its association with postoperative evolution. This protein shows a low expression in patients with complicated evolution.

EuroSCORE consists of a risk stratification model developed for predicting hospital mortality after cardiac surgery^[7-9]^, and it was an independent predictor of complicated evolution for this patient population. Several studies have demonstrated the validity of its use as a predictor of postoperative morbidity in cardiac surgery^[22,23]^ and the results of the present study are in agreement with the literature. The chance of complication increased by 2.09 times with each increased unit of EuroSCORE; values > 5 were predictors of complicated evolution in patients with normal ventricular function who underwent myocardial revascularization. EuroSCORE has also presented excellent sensitivity for the detection of complications in patients undergoing CABG in this population^[9,10]^. Present data confirm these findings, with a sensitivity of > 90%.

The arterial lactate level tended to be higher in the group with complicated evolution than in the group with uncomplicated evolution, although no statistically significant differences were observed. Approximately 10%-20% of patients present high lactate levels and postoperative morbimortality^[6]^. Although there was an increase in lactate levels in the group with complicated evolution, it was not consistent enough to be considered as an independent prognosis predictor. This absence of statistical significance may be explained by the limitation of the analysis, which was restricted to the intraoperative period. This period was too short to allow for arterial lactate clearance or to study its kinetics.

The results of this study suggest that HSP 70 is an independent uncomplicated evolution predictor after myocardial revascularization in patients with normal left ventricular function. This marker presented a sensitivity of > 93%, which constitutes a protection factor.

High levels of HSP 70 are related to lower damage caused by ischemia^[24]^. The presence of postoperative complication seems to be associated with low levels of HSP70. After inducing HSP 70 overexpression, Okubo et al. demonstrated a significant reduction in the infracted area^[24]^. The ischemic heart appears to be less capable of producing HSP 70, a factor which could be closely related to the decrease of the ability to tolerate stress after a surgery^[25]^. Experimental results suggest that the effect of moderate hypothermia during CPB involves the regulation of the HSP 70 protein family, with the inhibition of necrosis, but not of apoptosis^[19]^. HSP 70 levels have previously been associated with tissue damage and ischemia following cardiac surgery, and its measurement may be a diagnostic and prognostic advantage in these cases^[20]^.

The differences in HSP 70 expression and its correlation with postoperative evolution may be influenced by the groups, since ageing negatively regulate the HSP 70 expression in heart and can cause the heart to be more susceptible to oxidative and stress damage^[26]^. This can reinforce our argument that an increase in HSP 70 expression should be a cardioprotection mechanism, because the complicated evolution group showed higher age. The other possible influence was the group's sex prevalence. Sex hormone alters the expression of HSP 70 differently in women and men^[27]^. However, it's unclear if the men are more sensitive to induction of HSP 70 expression by the inhibitory effect of estrogen on the HSP 70 expression^[28]^ or if the women hormone doesn't have this inhibitory effect and other factors are involved in upregulation of HSP 70 expression^[29]^.

Circulating HSP 70 may indicate cellular stress or damage. Furthermore, HSP are suggested as immunoregulatory agents, and may be important in the host defense postoperatively^[30]^. The effects of two different cardioplegia techniques on myocardial HSP 70 messenger ribonucleic acid (mRNA) levels suggest that there was a significant increase in HSP 70 mRNA levels in response to CPB in both groups^[31]^. However, we didn't find a causal correlation between atrial and serum HSP 70 expression. The inductions of expression of this protein are tissue-specific and the circulation levels can be influenced by other origins. New study researches should explore if the increase in the HSP 70 cardiac expression may positively or negatively regulate the serum protein expressions.

The mechanism of HSP 70 expression preconditioning to increase success of cardiac surgery interventions is unclear but the present findings suggest that because HSP 70 levels are inherently high in patients with more favorable evolution and there is a correction with EuroSCORE, any protective benefit that can be derived from this protein can already be measured. This is in agreement with the literature^[32]^. The development of mechanisms that increase tolerance to perioperative damage may result in a more advanced method to attenuate CPB-induced inflammation and minimize this problem; measures that increase HSP 70 expression can be part of these mechanisms.

## CONCLUSION

The present findings demonstrate that some predictors, such as EuroSCORE, are independent complication predictors. Furthermore, the myocardial or serum HSP 70 levels can be considered as independent predictors of uncomplicated evolution in patients without ventricular dysfunction who undergo CABG, thereby providing additional information on the prognosis of these patients.

We conclude that the HSP 70, whether myocardial or serum (the latter being more feasible to measure), may be a relevant biomarker. A better understanding of the role of HSP in the inflammation caused by CPB may provide the possibility of predicting and helping prevent an adverse event in the immediate postoperative period following cardiac surgery.

It is believed that the identification of patients at high risk of complications during cardiac surgery using scores and known markers, associated with other predictors with strong predictive power, may allow for the adoption of early measures aimed at better tissue perfusion, resulting in a shorter ICU stay and better prognosis for patients undergoing myocardial revascularization.

**Table:** 

Authors' roles & responsibilities	
MAC	Design of the work; or the acquisition, analysis, or interpretation of data for the work; final approval of the version to be published
RMSA	Drafting the work and revising it critically for important intellectual content; final approval of the version to be published
PNM	Substantial contributions to the acquisition of data for the work; final approval of the version to be published
VASJ	Substantial contributions to the acquisition of data for the work; final approval of the version to be published
CSM	Substantial contributions to the acquisition of data for the work; final approval of the version to be published
JAF	Substantial contributions to the acquisition of data for the work; final approval of the version to be published
JLF	Substantial contributions to the acquisition of data for the work; final approval of the version to be published
PCBL	Design of the work; or the acquisition, analysis, or interpretation of data for the work; final approval of the version to be published
